# Neutron interference from a split-crystal interferometer

**DOI:** 10.1107/S1600576722006082

**Published:** 2022-07-15

**Authors:** H. Lemmel, M. Jentschel, H. Abele, F. Lafont, B. Guerard, C. P. Sasso, G. Mana, E. Massa

**Affiliations:** a ATI – Atominstitut, TU Wien, Wien, Austria; b ILL – Institut Laue–Langevin, Grenoble, France; c INRIM – Istituto Nazionale di Ricerca Metrologica, Torino, Italy; Tohoku University, Japan

**Keywords:** neutron interferometry, split-crystal interferometers, silicon crystals, atomic scale positioning

## Abstract

The first successful operation of a neutron interferometer with a separate beam-recombining crystal is reported. This result was achieved at the neutron interferometry setup S18 at the ILL in Grenoble by a collaboration between TU Wien, ILL, Grenoble, and INRIM, Torino. While previous interferometers have been machined out of a single-crystal block, in this work two crystals were successfully aligned on nanoradian and picometre scales, as required to obtain neutron interference. As a decisive proof-of-principle demonstration, this opens the door to a new generation of neutron interferometers and exciting applications.

## Abstract 2

This article reports the first successful operation of a neutron interferometer with a separate beam-recombining crystal. This result is a proof-of-principle demonstration showing that new-generation neutron interferometers and applications are possible.

## 1. Introduction

Neutron interferometry requires splitting and recombining the wavefunction of every single neutron while maintaining the coherence of the neutron wave packet. For thermal neutrons, a frequent approach is the use of silicon single crystals. Diffraction allows one to efficiently fulfil the task of splitting, reflecting and recombining the beams. The path separation is typically of the order of centimetres.

Since its first demonstration by Rauch *et al.* (1974 ▸), this technique has been used in a broad spectrum of fields (Rauch & Werner, 2000 ▸). Being based on the superposition of matter waves of a single electrically neutral particle with spin 1/2, neutron interferometry was used for a large number of critical tests of quantum mechanics, from the 4π symmetry of fermions (Rauch *et al.*, 1975 ▸) to the demonstration of the quantum Cheshire Cat (Denkmayr *et al.*, 2014 ▸). It was used to study the interplay with gravity in the famous Colella–Overhauser–Werner (COW) experiment (Colella *et al.*, 1975 ▸), to measure neutron scattering lengths (Haun *et al.*, 2020 ▸) and to search for the existence of hypothetical new forces (Lemmel *et al.*, 2015 ▸; Li *et al.*, 2016 ▸).

Achieving neutron interference requires the alignment of the involved crystal planes within a few nanoradians. Relative displacements of more than a few picometres must be avoided. Up to now, these problems have been solved by machining the entire interferometer out of a monolithic single crystal. This design – based on the availability of perfect-single-crystal ingots – has limitations: the sensitivity of many experiments depends critically on the area enclosed by the two wave paths and therefore by path separation and path length within the interferometer. Another limitation is the size and complexity of test objects that can be inserted into the interferometer. Finally, the sensitivity is significantly impacted by the uniformity of the lattice spacing in the diffracting crystals (Heacock *et al.*, 2017 ▸). Large single-ingot interferometers (Zawisky *et al.*, 2009 ▸) showed difficulties associated with strain fields within large crystals.

A promising solution to dramatically improve the sensitivity of neutron interferometers would be an interferometer consisting of separated crystals. An attempt to build such a split-crystal interferometer is reported by Uebbing (1991 ▸), but it did not succeed in achieving neutron interference. Neutron interferometry with physically split gratings using cold or very cold neutrons is reported by Zouw *et al.* (2000 ▸), Pruner *et al.* (2006 ▸) and Sarenac *et al.* (2018 ▸). However, the small separation of the beam paths and/or the low count rates of very cold neutron beams did not allow competitive sensitivity.

Perfect-crystal interferometers are also used with X-rays, as first demonstrated by Bonse & Hart (1965 ▸). In contrast to neutrons, X-rays are substantially absorbed by the crystal. While the loss of intensity is compensated by brighter sources, the absorption creates wider beam acceptance angles. It was therefore already possible in 1968 to create a split-crystal interferometer for X-rays (Bonse & te Kaat, 1968 ▸; Deslattes, 1969 ▸).

**Figure 1:**
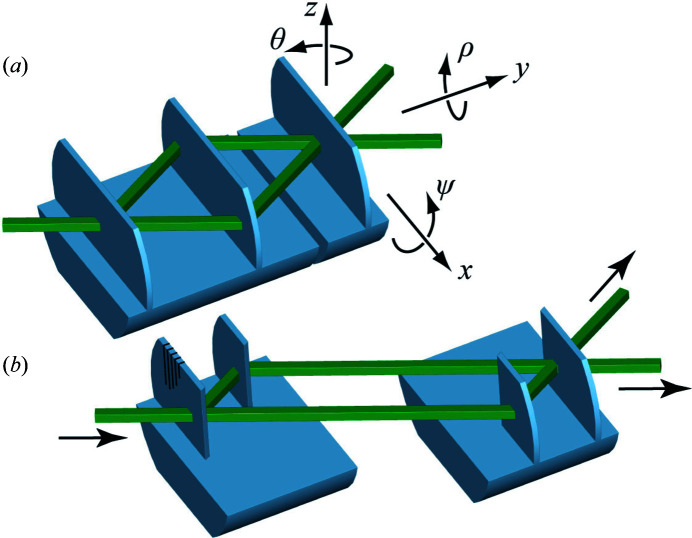
(*a*) Symmetric and (*b*) skew-symmetric crystal interferometers. The neutrons are coherently split, reflected and recombined by the crystal lamellas. The two crystals of a skew-symmetric interferometer can be placed far apart.

In the case of a symmetric interferometer, the third lamella (the analyser) is separated [*cf*. Fig. 1 ▸(*a*)]. Then the interference signal is extremely sensitive to the analyser’s *x* position, since a movement by one lattice constant (*d* ≃ 0.192 nm) creates a phase shift of 2π. Such an interferometer has allowed measuring the lattice parameter of ^28^Si with parts per billion accuracy (Massa *et al.*, 2011 ▸, 2015 ▸), leading to the determination of the Avogadro constant (Fujii *et al.*, 2018 ▸) and thereby realizing the kilogram by counting atoms (Massa *et al.*, 2020 ▸).

Alternatively, two pairs of crystal lamellas can be separated, as shown in Fig. 1 ▸(*b*). These skew-symmetric split crystals are insensitive to misalignments along the *x* axis and allow the realization of long and spaced interferometer arms, as well as scans of the arm length and Bragg angle alignment. They have been used for phase-contrast imaging (Yoneyama *et al.*, 2002 ▸), profiting from the extended sensed area and volume.

This article reports on the first successful operation of a split-crystal interferometer using thermal neutrons, showing that all requirements to build such a device are under control. We used an existing symmetric split-crystal interferometer, but the proof-of-principle demonstration is just as valid for a skew-symmetric setup.

## 2. Alignment requirements

**Figure 2:**
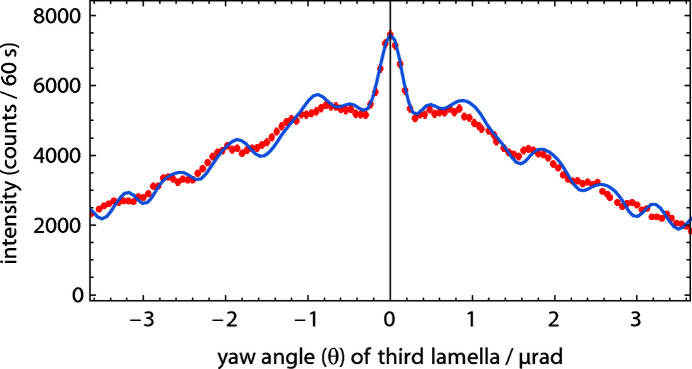
A rocking curve of the analyser lamella with path 1 of the interferometer blocked, *cf*. Fig. 3 ▸. The error bars lie within the bullets. The theoretical prediction (solid line) is calculated by convolving the product of three Bragg reflections (the triple-bounce Bragg monochromator) and two Laue reflections (the splitter and mirror lamella) with the final Laue reflection by the analyser. The overall agreement is very good. We attribute the remaining discrepancies to imperfections of the crystal geometries on the level of a few micrometres.

Neutron interference requires that the relative alignment of the two crystals of a symmetric interferometer fulfils the following conditions:

(i) θ, yaw angle, rotation about the vertical axis. This angle must be adjusted to match the Bragg condition. The observed triple-Laue rocking curve is shown in Fig. 2 ▸. The broad peak is modulated by Pendellösung fringes, which culminate in a central spike (Petrascheck & Rauch, 1984 ▸). While X-ray interference is possible within the whole broad peak, neutron interference is only possible in the central spike (Mana & Vittone, 1997 ▸) which has a full width at half-maximum of ∼250 nrad.

(ii) ρ, pitch angle, rotation about the lamella’s surface normal. This angle must be adjusted to make the interfering beams parallel. If misaligned, the beams are slightly inclined to each other and create a horizontal moiré pattern. To make the interference observable, the fringe spacing Λ_*z*_ = *d*/ρ must be greater than the vertical detector resolution, where *d* = 0.192 nm denotes the spacing of the diffracting {220} planes of the silicon crystals. The alignment of the ρ angle is not trivial because – in contrast to the θ angle – its variation hardly changes the intensity. Our alignment strategy is described below.

(iii) ψ, roll angle, rotation about the diffracting-plane normal. No accurate adjustment is required. The interferometer is insensitive to this misalignment unless it becomes macroscopic.

(iv) *x*, axial position along the diffracting-plane normal. Statically, this degree of freedom is unessential, but the noise or drift during the measurement time must be less than a few picometres, since a displacement of the analyser by one diffracting plane creates a full interference period. This applies only to a symmetric interferometer. In the skew-symmetric layout, all translational degrees of freedom cancel, making it the best choice for a large-scale interferometer.

(v) *y*, transverse position along the lamella’s surface normal. To avoid defocusing, the analyser-to-mirrors distance must be equal to the splitter-to-mirrors distance to within a few micrometres. This applies only to a symmetric interferometer. In the skew-symmetric layout, the two crystals can be spaced at will.

(vi) *z*, vertical position. X-ray and neutron propagations are unaffected by a vertical shift of the interferometer crystals. No adjustment is required.

(vii) The lattice constants of the two crystals must be equal, otherwise vertical moiré fringes occur, which are spaced by *d*/ε_xx_, where ε_xx_ = αΔ*T* is the thermal strain between the crystals. Taking into account the thermal expansion of silicon, α = 2.5 × 10^−6^ K^−1^, this means that the temperature difference Δ*T* of the two crystals must be less than 10 mK.

All these parameters must not only be aligned but also be kept constant over a typical measurement time. Alternatively, if a parameter is drifting but can be monitored, time-dependent neutron detection can be used to reconstruct the correct phase.

## 3. Experimental setup

**Figure 3:**
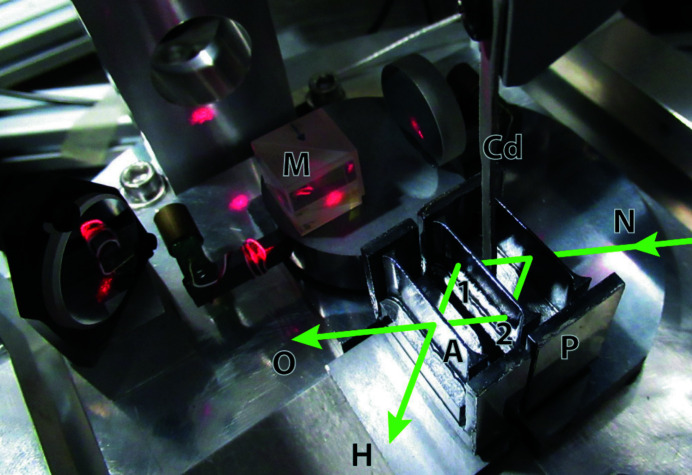
The crystal interferometer with separate analyser lamella (A). The neutron beam (N) enters from the right side, is split into two paths (1, 2), and exits through the output ports O and H. Polished surfaces on the crystal’s sides allow pre-alignment by an optical autocollimator on the front (P) and monitoring of the analyser’s coordinates (θ, ρ and *x*) by an optical interferometer (M) on the rear. A piece of cadmium (Cd) can be inserted from the top to block one or the other interferometer path.

The setup is depicted in Fig. 3 ▸. We used an existing symmetric X-ray interferometer (Ferroglio *et al.*, 2008 ▸) manufactured originally by INRIM to measure the Si lattice parameter. The splitter and mirror lamellas are monolithically connected while the analyser lamella is separate. The lamellas are ∼0.73 mm thick and have a spacing of ∼10 mm. The analyser is mounted on a piezo-driven tip–tilt platform developed earlier by INRIM (Bergamin *et al.*, 2003 ▸), which allows one to vary both pitch and yaw angles by ∼±70 µrad with sub-nanoradian resolution. We added another piezo stage to vary the axial position of the analyser by a few micrometres with picometre resolution and integrated it into a small platform that hosts the laser interferometer for monitoring the analyser position and pitch and yaw misalignments.

The two crystals sit on silicon supports and are held in position by thin films of high-viscosity silicon oil. We arranged them with micrometre accuracy using a homemade coordinate-measuring machine, which was then removed from the setup. Both crystals have optically polished surfaces on their sides with identical relative orientation to the lattice planes. To pre-align the split crystals, we made these surfaces parallel to each other within one arcsecond using an optical autocollimator. An optical interferometer used the opposite polished surface to monitor the analyser’s θ and ρ angles and the *x* axial position, allowing for a closed-loop operation.

In the forward exit beam we used a multi-channel neutron detector recently developed by the ILL. It is a flat ^3^He detector with 90% efficiency and 1 mm spatial resolution achieved by crossed wire electrodes. Details of this detector type are given by Buffet *et al.* (2017 ▸).

A vibration-isolated bench supports the monochromator crystal, which is located in a thermal neutron beamline, and the optomechanical mounts of the interferometer. To ensure temperature stability, the bench is surrounded by two thermal housings.

An aperture in front of the interferometer reduced the beam size to 1 mm in width and 8 mm in height, which was the maximum the interferometer could accept (having been designed for X-ray operation). A horizontal slit ∼1 m upstream controlled the vertical divergence. A slit of 2 mm in height left a mean intensity in the two exit beams of 48 counts per second. A 20 mm slit delivered 280 counts per second. The background amounted to 0.31 counts per second. We used a neutron wavelength of λ = 0.19 nm, which corresponds to a Bragg angle of 30° on our silicon {220} diffracting planes. The wavelength distribution was δλ/λ ≃ 1.7%.

## 4. Results

**Figure 4:**
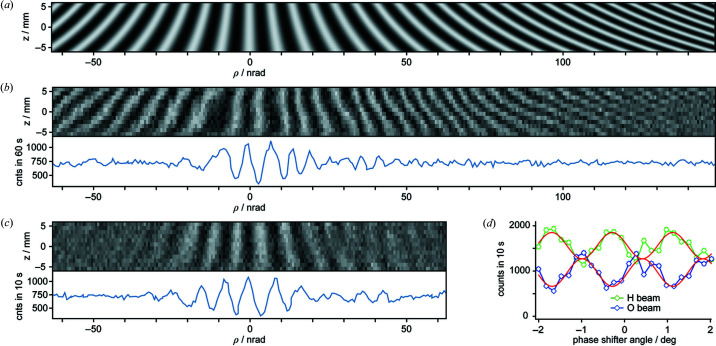
(*a*) Theoretical and (*b*) observed interference generated during the ρ angle alignment. For each ρ value, a neutron-camera image is horizontally integrated yielding a vertical intensity distribution, which is shown here by each pixel column. Concatenating the columns of all ρ values generates the complete image (*b*). The theoretical pattern (*a*) perfectly reproduces the observed pattern, although the vertical fringes fluctuate in position and spacing due to phase drifts during the measurement. (*c*) If the full-beam height is used, the interference is visible only in the vicinity of perfect ρ alignment. The bottom figures in (*b*) and (*c*) show the total camera counts. (*d*) An interferogram created with an auxiliary phase shifter. The red curves are the best sinusoids fitting the data. The fringe visibility is ∼40%.

Fig. 4 ▸(*b*) shows the interference pattern observed during the alignment of the ρ angle. For this plot, the ρ angle has been scanned in steps of 0.65 nrad and the detector images have been integrated horizontally, leaving a one-pixel column for each ρ value. These columns are plotted next to each other, creating a pattern that originates from two effects.

Firstly, if the ρ angle is misaligned, horizontal moiré fringes develop, because the two interfering beams are vertically inclined to each other. As ρ = 0 rad is approached, the fringe spacing becomes larger and larger until the phase is uniform over the whole beam height. The fringe spacing is given by Λ_*z*_ = *d*/ρ if plane waves are assumed. For a divergent beam originating from a point source, the spacing is magnified by *l*_D_/*l*_A_E (∼1.55 in our case), where *l*_D_ is the distance between source and detector, and *l*_A_ is the distance between source and analyser lamella.

The second effect originates from the fact that the centre of the ρ rotation is *z*_0_ = 38 mm below the neutron beam. Therefore, each ρ change displaces the analyser along the *x* axis and causes a phase shift that is visible as vertical fringes superimposed on the horizontal ones.

Combining both effects, we expect the interference pattern 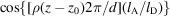 , which is plotted in Fig. 4 ▸(*a*). The observed pattern in Fig. 4 ▸(*b*) reproduces the horizontal fringes exactly. The vertical fringes have slightly irregular spacings and positions because the analyser *x* position and, consequently, the fringe phase were drifting during the five-hour measurement time.

Fig. 4 ▸(*c*) shows the same pattern with the horizontal slit fully open, *i.e.* with the interferometer illuminated by an incoherent array of point sources. In this case, the horizontal moiré pattern, expected in regions of misaligned ρ angle, disappears. Although each point source creates its own interference pattern, each pattern is vertically shifted and, in the mean, averaged out. Therefore, whenever an extended source (like a neutron source) is used, the interference is only visible in the vicinity of the ρ = 0 rad alignment. This applies in any case if spatially integrating detectors are used, as shown in the bottom parts of Figs. 4 ▸(*b*) and 4 ▸(*c*).

The motivation of this proof-of-principle experiment was twofold. Firstly, we wanted to understand if the crystal alignment was possible using neutron detection only. For this task, we identified an efficient procedure based on the pattern described above. The pre-alignment of the ρ angle, either by using the autocollimator or by measuring neutron intensities, leaves a range of a few microradians, which has to be searched for the 40 nrad wide spot of interference. While scanning the ρ angle, we continuously applied a fast Fourier transform algorithm to a moving time window of (spatially integrated) neutron counts (Andreas & Kuetgens, 2020 ▸). Being sensitive to periodic modulations of the counts, we could find the correct alignment even if the measurement time per ρ step was so short that the interference amplitude was of the order of the statistical fluctuations. This way we could substantially speed up the ρ scanning.

Secondly, we needed to check the level of the environmental seismic and acoustic noise, as well as their effect on a split-crystal interferometer. The picometre-scale alignment of the two crystals is demonstrated by the neutron interference. In fact, owing to the long neutron-count times, typically 10 s, any high-frequency noise exceeding some tens of picometres would wipe out the interference.

To produce a conventional interferogram, the natural choice would be a controlled *x* motion of the analyser lamella. Unfortunately, operation in air jeopardized satisfactory closed-loop operation. In fact, the fluctuating index of refraction limited the stability of the optical interferometer, inducing noise onto the crystal position rather than stabiliz­ing it.

**Figure 5:**
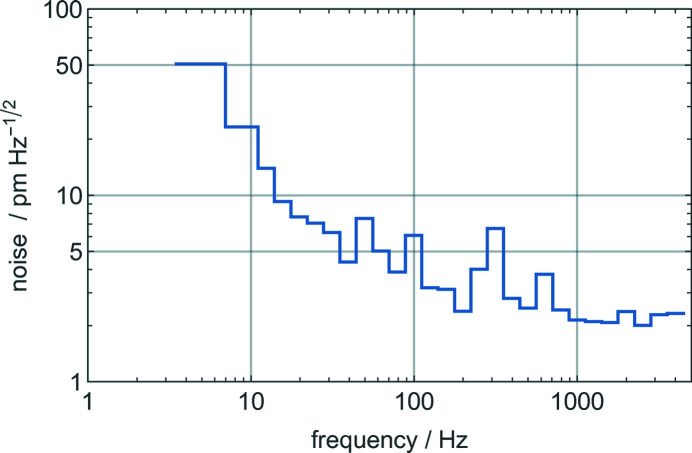
Amplitude spectral density of the analyser position noise. The abscissa is gauged in one-third of octave frequency bands.

We quantified the noise by looking at the signal of the optical interferometer; the amplitude spectral density (*i.e.* the square root of the power spectrum) of the position noise is shown in Fig. 5 ▸ over a 4 kHz bandwidth. Expected peaks are observed at 50 and 100 Hz; the broad peak at ∼300 Hz might originate in the mechanical resonances of the tip–tilt platform supporting the analyser (Bergamin *et al.*, 2003 ▸). The 1/*f* rise of the low-frequency noise originated from fluctuations of the refractive index of the air (which do not affect the open-loop operation of the interferometer) and mechanical and thermal instabilities of the crystal alignment. Looking at future skew-symmetric realizations, operation in vacuum and optoelectronic feedback of the alignment errors will suppress it.

Eventually, all measurements were made in open-loop mode, and, to scan the interference fringes, we introduced a conventional phase shifter made of a 4 mm thick aluminium slab between the second and third lamella. The scan result is shown in Fig. 4 ▸(*d*). The expected sinusoidal modulation is disturbed by strong fluctuations that we attribute to low-frequency position noise of the analyser. Nevertheless, we achieved an interference contrast of ∼40%.

## 5. Outlook

We have demonstrated that the alignment of two crystals with the accuracy required for neutron interference is technically possible. This result is crucial for the development of a split-crystal interferometer with a skew-symmetric geometry and will drive its commissioning.

Contrary to the symmetric interferometer, the skew-symmetric geometry makes the interferometer insensitive to the relative displacement of the crystals. The requirements for the environmental (seismic and acoustic) noise and the alignment of the yaw and pitch angles are similar. However, since the fringe phase is proportional to the displacements of the splitter and analyser versus the mirrors, it is sensitive to the yaw rotation between the crystals. Typically, with 0.2 m arm separation, 1 nrad rotation scans a full interference period.

Therefore, a laser interferometer must be coupled to the split crystals for precision measurements of their relative Bragg and pitch angles. An example is γ-ray diffractometry, which also requires high-resolution angle measurements (Krempel, 2011 ▸). Operation in a vacuum would boost its stability and allow a closed-loop operation based on the feedback of the signals. Active control of the crystals’ temperature will be necessary to keep it uniform to within the required 10 mK. Eventually, the simultaneous operation of the crystal interferometer with X-rays could monitor systematic errors, *e.g.* due to non-uniformities of the lattice constant.

The split crystals of a skew-symmetric interferometer can be placed far apart. Therefore, long arms are possible and their length can even be varied. We see a large potential to achieve reliable and robust phase measurements, even with crystal separations up to the metre scale. Large-scale split-crystal interferometers will open the way to several experiments testing fundamental symmetries and interactions, in particular exploring quantum mechanics and its relation to gravity. The first experiments, profiting from the increased area enclosed by the neutron paths, could be more sensitive repetitions of the COW experiment, studying the weak equivalence principle in the quantum regime (Saha, 2014 ▸). Experiments on hypothetical new forces are important tools for probing dark energy fields (Lemmel *et al.*, 2015 ▸; Li *et al.*, 2016 ▸). Introducing massive test masses into the interferometer will allow neutron tests of gravitomagnetism (Hammad *et al.*, 2021 ▸).

Our result is a proof of principle showing that such experiments can be realistically considered. Because of the complexity of the project, it is difficult to predict the interferometer performance, but we do not see principle limitations.
